# Shape Memory Hydrogels for Biomedical Applications

**DOI:** 10.3390/gels10040270

**Published:** 2024-04-17

**Authors:** Aleeza Farrukh, Sana Nayab

**Affiliations:** 1 Department of Chemical and Biomolecular Engineering, University of California, Irvine, CA 92697, USA; 2 Institute of Chemistry, Quaid-i-Azam Campus, University of the Punjab, Lahore 54590, Pakistan

**Keywords:** shape memory hydrogels, stimuli-responsive polymer, reconfigurable biomaterials, drug delivery, soft actuators, smart tissue engineering scaffolds

## Abstract

The ability of shape memory polymers to change shape upon external stimulation makes them exceedingly useful in various areas, from biomedical engineering to soft robotics. Especially, shape memory hydrogels (SMHs) are well-suited for biomedical applications due to their inherent biocompatibility, excellent shape morphing performance, tunable physiochemical properties, and responsiveness to a wide range of stimuli (e.g., thermal, chemical, electrical, light). This review provides an overview of the unique features of smart SMHs from their fundamental working mechanisms to types of SMHs classified on the basis of applied stimuli and highlights notable clinical applications. Moreover, the potential of SMHs for surgical, biomedical, and tissue engineering applications is discussed. Finally, this review summarizes the current challenges in synthesizing and fabricating reconfigurable hydrogel-based interfaces and outlines future directions for their potential in personalized medicine and clinical applications.

## 1. Introduction

Shape memory polymers (SMPs) have emerged as an important class of materials due to their ability to change shape in response to a specific stimulus, making them exceedingly useful in various areas, including tissue engineering, drug delivery, soft robotics, and flexible bioelectronics [1,2,3,4,5]. Initially, polycaprolactone (PCL)-based SMPs showed potential in biomedical applications, leading to progress in the fabrication and engineering of a range of biocompatible shape memory materials [5]. Consequently, shape memory hydrogels (SMHs) have been receiving a lot of attention due to their inherent biocompatibility, programmable shape-morphing capabilities, tunable physiochemical properties, and controllable biodegradability [6,7,8,9]. Therefore, SMHs are promising materials for biomedical applications because of their outstanding features, including high stretchability, transparency, ionic conductivities, wettability, and biocompatibility [10,11,12,13]. 

SMHs provide innovative capabilities for biomedicine based on their unique responsiveness to a wide range of biocompatible stimuli (e.g., thermal, chemical, electrical, light) under physiological conditions. For example, SMHs are reported for controlled drug delivery by modulating the volume ratios of the polymers, as stents/catheters for surgeries by reconfiguring the polymeric network, and as implantable scaffolds for regenerative therapies by relaxation of internal structure into programmed dimensions [7,11,14,15]. Moreover, the biofunctionalization of SMHs with different cell-adhesive or cell-signaling biomolecules enhances their biocompatibility and supports the growth and differentiation of cells in biological tissues [10,16,17]. In turn, SMHs have been targeted for use in biomedical applications, including drug delivery, surgical sealants, stents, tissue engineering, regenerative therapies, and biosensing [18,19,20].

The next sections discuss the fundamental working mechanism of SMHs, types of SMHs based on the applied stimuli, and their use in different biomedical applications. These sections are organized to understand the shape morphing of SMHs in response to various stimuli. In the first section, the fundamental working mechanisms and generalized programming strategies for SMHs are discussed. In the second section, the recent literature is structured to highlight the development of SMHs by considering (i) the chemical structure of the core polymeric networks, (ii) the dynamic physical and chemical interactions of the polymeric matrixes, (iii) the strategy for programming SMHs, and (iv) the externally stimulated shape morphing of SMHs. The third section of this review provides examples of SMHs for surgical, biomedical, and tissue engineering applications. Finally, this review summarizes the overall progress in the engineering and development of reconfigurable hydrogel-based interfaces and outlines future directions for their potential in personalized medicine and clinical applications.

## 2. Fundamental Mechanism and Programming of Shape Memory Hydrogels

**Figure 1:**
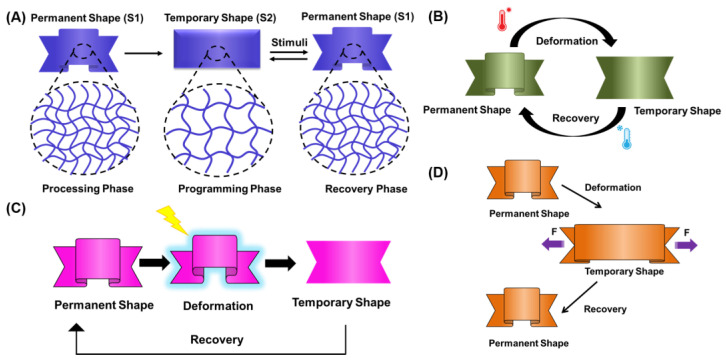
(**A**) Illustration of the working mechanism of SMHs, where the initial permanent (crosslinked network) shape (**S1**) obtained during the *processing phase* is deformed into a temporary (flexible network) shape (**S2**) during the *programming phase*. Such a temporary shape of polymer recovers back to its initial shape (**S1**) in the presence of external stimuli. (**B**) An illustration of the thermal processing of SMHs by heating or freeze-thawing the polymeric network into a desired permanent shape. (**C**) An illustration of the light-mediated processing of SMHs, where photopolymerization or stereolithography techniques are employed to obtain polymeric networks with a desired permanent shape. (**D**) An illustration of the mechanical deformation processing of SMHs, where polymeric networks with desired permanent shapes are obtained with stretching, molding, and extruding methods.

SMHs have at least two types of polymeric networks: the permanent network, responsible for the initial shape (**S1**) of the hydrogel, and the transient network, which allows for the transition to a temporary shape (**S2**) in response to an external stimulus (Figure 1A). In contrast to traditional responsive polymers (thermal, light, etc.), typically, shape morphing is not an inherent property of the polymers and instead is programmed during the fabrication process [7,21,22]. Therefore, SMHs are highly deformable materials that can be programmed to recover to a memorized shape in response to environmental changes by modulating their physical structure [7,23]. This fundamental mechanism of phase separation differentiates the shape transformation of SMHs from typical responsive polymers. 

The programming of SMHs encompasses different phases. First, in the *processing phase,* SMHs are processed into an initial permanent (rigid network) shape (**S1**), followed by the *programming phase*, where the processed polymers are deformed (softer network) and fixed into a temporary shape (**S2**). Last, in the *recovery phase,* the polymers can revert back to their initial shape (**S1**) in the presence of external stimuli (Figure 1A) [6,24]. The *programming phase* and *recovery phase* of SMHs can be sequentially switched on and off multiple times with external stimulation. The ordered state of SMHs corresponds to the chemical structure or physical crosslinking that sets the initial/permanent shape (**S1**) of the polymer [1,25]. The disordered structure state is attributed to flexible and reversible chains that allow for switching and recovery to a temporary shape (**S2**) [25,26].

Shape memory polymers are typically processed using thermal treatment above the LCST of the polymer, mechanical deformation (stretching, molding, and extruding), or stereolithography (Figure 1B–D). During processing, SMHs are typically deformed into an initial permanent shape by applying an external force when the temperature is above the glass transition (Tg) or melting temperature (Tm) of the polymers [25,26,27]. At these temperatures, the molecular chains of polymers are flexible and can be molded into the desired shapes or dimensions. Upon cooling below Tg or Tm, the flexible chains become rigid while storing the memory of stress in the molecular structure. During programming phase, the polymer sample is deformed and fixed into a temporary shape, which, upon external stimuli, resumes the original positions of the crosslinked network and releases stored stress [21,22,28]. The different phases of SMHs are typically stabilized by dynamic covalent bonds or electrostatic interactions between polymeric networks that during shape morphing break and reform bonds between adjacent polymer chains, providing mechanical stability to both shapes [21,27]. Analogously, SMHs programmed by external mechanical deformation can recover to their initial shape in the presence of external stress [1,29,30].

Recently, the programming phase of SMHs is also carried out by cooling below the critical temperature (Tc) or by tuning the dynamic crosslinking of the polymeric networks. Such processing not only allows for faster recovery of SMHs but also allows for shape morphing under physiological conditions [19,25,29]. SMHs can be fabricated in different forms and dimensions, including thin films, micro- or nanofibers, 3D hydrogels, or 4D-printed scaffolds [21,23,25].

## 3. Types of Shape Memory Hydrogels

The different types of SMHs are classified based on the external stimuli that control the actuation mechanism. In this regard, different external stimuli can be applied to trigger shape morphing, including heat [31]; light [32]; moisture [33]; pH [34]; and mechanical [35], electrical [36], and magnetic stimuli [37]. This section encompasses different types of SMHs classified by actuation mechanisms and external stimuli [38]. Moreover, a detailed table (Table 1) is included to illustrate various chemical and physical crosslinking strategies employed to modify the established polymeric hydrogel networks (AAm, PEG, NIPAM, etc.) into various SMHs. Subsequently, to facilitate classifications of SMHs, both from fundamental chemistry to an actuation mechanism, the role of additives (i.e., ions, nanofillers, hybrid polymers) in obtaining SMHs with various stimuli-responsiveness is also summarized in Table 1. Finally, to summarize the fabrication and processing strategies to develop functional interfaces from SMHs, a table (Table 2) containing representative examples from different types of SMHs is also included.

### 3.1. Thermally Responsive SMHs

**Figure 2:**
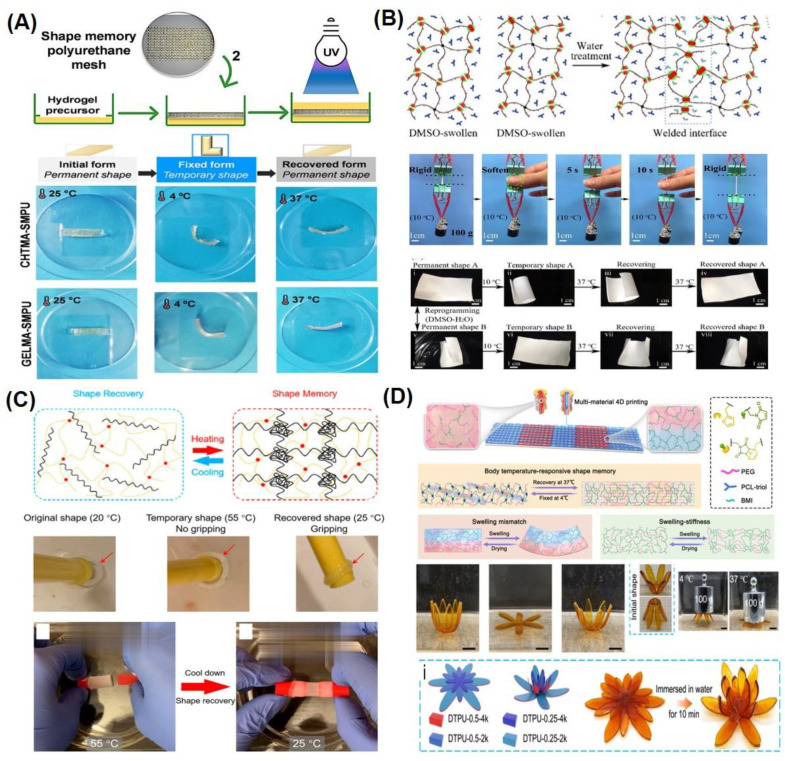
Representative examples of thermally responsive SMHs. (**A**) Schematics for preparation of PU-based SMHs, where the thermo-responsive PU layer was sandwiched between various non-responsive biopolymers (top), with digital camera images of shape-morphing stages of such PU composite with chitosan methacrylate and gelatin methacrylate at 37 °C (bottom). Reproduced with permission from the American Chemical Society [44]. (**B**) Schematics for preparation of P(AAm)-based SMHs programmed by physical cross-linking of polymeric networks on the exchange of DMSO with water (top), with digital camera images of shape-morphing stages of such P(PEA-co-AAm) hydrogel sheets at 37 °C (bottom). Reproduced with permission from the American Chemical Society [46]. (**C**) Schematics for the programming of ELP-based SMHs with a desired shape at 55 °C and recovery of the original shape at 20 °C (top), with digital camera images of shape-morphing stages such P(AAm) and ELP-based dual network hydrogels at 25 °C (bottom). Reproduced with permission from the American Chemical Society [47]. (**D**) Schematics for the multi-material 4D printing process of dynamic thermoset polyurethanes (DTPU) extruded to fabricate a swelling mismatch scaffold with body temperature-responsive shape memory, where water triggered programmable deformation (top), with digital camera images of shape-morphing stages of the DTPU scaffold at 37 °C. Reproduced with permission from Springer Nature Publishing Group [50].

Thermally responsive SMHs can be programmed to actuate when stimulated by an external thermal stimulation. Thermal activation was the first reported means of externally actuating SMPs and is still the most widely used shape-morphing approach [39,40,41,42]. Typically, thermoresponsive materials are prepared by functionalization/modification of thermoresponsive polymeric networks to allow for temporal shape-morphing. Towards this end, polyurethane (PU)-based polymers were employed for the fabrication of thermoresponsive SMHs, where fabricated PU fibers were programmed from a straight permanent shape to a hooked temporary shape upon processing over a temperature range. These fibers undergo shape morphing on thermal activation and are demonstrated for neural stimulation in nerve regeneration therapy [43]. A broadly applicable strategy to develop PU-based SMHs that exhibit fast shape recovery ratios (~100%) at body temperature (37 °C) is by embedding a thermoresponsive PU mesh sandwiched between non-thermoresponsive biopolymeric hydrogel networks such as methacrylated chitosan (CHTMA), gelatin (GELMA), laminarin (LAMMA), or hyaluronic acid (HAMA). These layer-by-layer hydrogels were programmed by thermal molding (at 37 °C or 65 °C) and fixation (at 4 °C) while displaying shape morphing under the physiological range (37 °C) (Figure 2A) [44]. 

Recently, dual network hydrogels have been widely reported for thermoresponsive SMHs, where the stimuli-responsive polymeric network is stabilized by a non-stimuli-responsive network. To this end, supramolecular hydrogels fabricated from widely biocompatible poly(ethylene glycol) (PEG) were reported using polyaddition polymerization with methylenediphenyl 4, 4-diisocyanate (MDI), and imidazolidinyl urea (IU). These hydrogels were stabilized by hydrogen bonding interactions of urethane groups as well as by the hydrophobic interaction of the benzyl ring. These SMHs undergo thermoresponsive shape morphing between 4 °C and 50 °C and were tested for postoperative peritoneal adhesion [45]. Similarly, polyacrylamide (PAAm)-based dual networks were reported by copolymerization of AAm with 2-phenoxyethyl acrylate (PEA), using DMSO as solvent. The replacement of DMSO from the prepared copolymer by water allowed for crosslinking of the copolymer promoted by PEA-mediated hydrophobic interactions leading to the formation of the hard network at 10 °C while undergoing morphing at body temperature (37 °C), as demonstrated by the softening of the polymer by pinching between two fingers (Figure 2B) [46]. Similarly, dual-network SMHs were also fabricated from photopolymerized P(AAm) and elastin-like polypeptides (ELP). These hydrogels were programmed into their temporary shape at the LCST of a physically crosslinked ELP network at 55 °C while undergoing fast (2–3 s) shape recovery at 20 °C (Figure 2C) [47].

Interestingly, dual networks were demonstrated for polyvinyl alcohol (PVA)-based biodegradable hydrogels by incorporation of cornstarch (CS) with a disulfide polyurethane crosslinker. These hydrogels were programmed into a temporary phase by drying the SMHs and undergoing shape morphing when placed in water [48]. Similarly, PVA-based thermoresponsive hydrogels were developed by blending with cationic chitosan and graphene oxide (GO) in water and glycerol. By taking advantage of PVA thermoresponsive behavior, these SMHs were programmed in a temporarily deformed shape using freeze-thaw cycles by cooling down to −20 °C. The PVA formed hydrogen bonds with glycerol, while cationic chitosan and GO were crosslinked through electrostatic interactions, forming an interpenetrating network (IPN). These hydrogels show shape recovery when immersed in water at 60 °C, demonstrated for strain sensing applications [40]. Moreover, the thermoresponsive hydrogels can be prepared by bioconjugation chemistries, such as thiol-ene or Diels–Alder reactions. For this purpose, thiolene/acrylate-based SMHs were reported, which were further coated with iridium oxide (IrO_2_) to improve electrochemical performances. The fabricated hydrogels were thermally programmed into a hook shape (50 °C and 0 °C) while undergoing shape morphing at 38 °C during in vivo nerve stimulation application [49].

Body-temperature-triggered SMHs are gaining attention for biomedical applications. For example, SMH was fabricated from reversible hydrophobic dipole pairing microdomains in the flexibly crosslinked network of AAm (acting as a hydrogen-bonding monomer), acrylonitrile (AN) (acting as a dipole monomer), and a long flexible PEG-MA crosslinker, followed by solidification by using BaSO_4_. This hydrogel demonstrated interesting mechanical properties comparable to those of rubbers while undergoing reversible shape morphing by adjusting temperatures ranging from 20 °C to 40 °C, which allowed for their use in in vivo transcatheter arterial embolization [51]. Similarly, PU-based hydrogels containing PEG, imidazolidinyl urea (IU), and methylene diphenyl 4,4-diisocyanate (MDI) formed by polyaddition reaction. These dynamic hydrogen bonds containing SMHs undergo shape morphing in physiological conditions (37 °C, 95% humidity) and allow for the controlled release of tannic acid (TA) and kartogenin (KGN) to promote MSC differentiation into chondrocytes [52]. Additionally, a body-temperature-responsive polymeric network was developed from PCL-triol, PEG, and Diels–Alder (DA)-diols consisting of N,N’-4,4′-diphenylmethane-bismaleimide and furfuryl alcohol, which act as chain extenders in DA-cycloaddition. These 4D hydrogels consisted of highly swellable 2D-printed polymeric meshes from PCL-triol and PEG, while a low swellable polymeric mesh was fabricated by incorporation of PCL-triol, PEG, and DA-diols. These networks were programmed to deform in water, forming a 1D mesh, while undergoing shape recovery at body temperature (37 °C) (Figure 2D) [50]. Despite this progress in the development of body temperature SMHs, very few room-temperature SMHs are reported. In recent efforts, naturally recoverable SMHs at room temperature were developed from photopolymerized poly(acrylic acid) (pAA) polymer by altering the degree of phase separation during the device programming phase. In this strategy, the developed 4D printable SMHs show different shape-shifting kinetics during phase separation by internal mass diffusion at ambient temperature [53].

Thermoresponsive SMHs are also finding great potential for the development of hydrogel actuators. To this end, thermoresponsive hydrogel actuators were demonstrated from bilayers composed of poly(N,N-dimethyl acrylamide-co-stearyl acrylate) (P(DMAAm-co-SA). These 3D-printed bilayer hydrogels undergo reversible shape morphing in water upon heating at different temperatures (50 °C to 70 °C) [54]. Despite the multiple approaches for the fabrication of thermoresponsive SMHs, there are not many approaches to generate SMHs with precisely controllable stepwise actuation. Accordingly, few approaches are reported to develop precisely controlled SMHs with the inclusion of liquid–crystalline (LC) materials. For example, the LC-vitrimer-based 3D actuators were developed using aza-Michael addition and photopolymerization between nematic mesogens (1,4-bis-[4-(3-acryloyloxypropyloxy) benzoyloxy]-2-methylbenzene) and primary (amine n-butylamine, n-BA). The use of such LC actuators allows for actuation stability in the nematic phase to facilitate step-wise shape morphing over a broad temperature range of 70 °C (i.e., from 35 °C to 105 °C) [55]. 

### 3.2. Chemically Responsive SMHs

**Figure 3:**
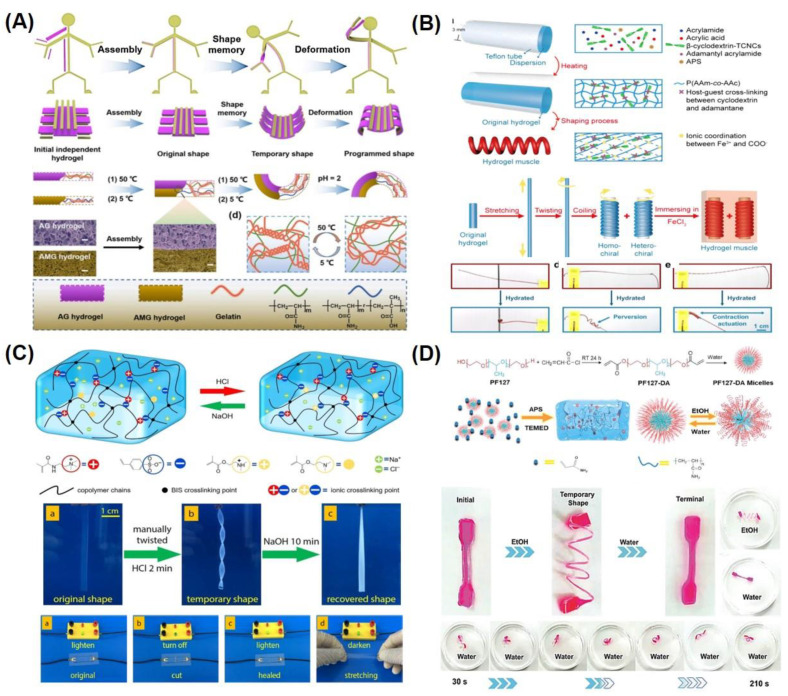
Representative examples of chemically responsive SMHs. (**A**) Schematics of shape morphing of hydrogel actuator (robot) (top) and the mechanism for formation of such hydrogel robot from IPNs of P(AAm)/gelatin (AG) and P(AA-co-MAA)/gelatin (AMG), undergoing shape morphing at pH 2 (bottom). Reproduced with permission from the American Chemical Society [58]. (**B**) Schematic diagram showing the fabrication process and network structure of the tendril-like artificial muscle fabricated from a terpolymer of AAm, AA, and Ad-Am, where the structure was locked by immersing in an FeCl_3_ solution (top), with digital camera images of different shape-morphing stages of such homochiral hydrogel muscles in response to water spray (bottom). Reproduced with permission from the American Chemical Society [61]. (**C**) Schematics of the cross-linking evolution of polyampholyte hydrogels fabricated from DMAEMA, MPTC, and NaSS (top), with digital camera images of different shape-morphing stages of such polyampholyte in response to a basic pH (bottom). Reproduced with permission from the American Chemical Society [63]. (**D**) Schematic illustration for the preparation of PLU-based hydrogels, where the structure of PLU micelles change in water (top), with digital camera images showing shape morphing of such copolymers of PLU-DA and P(AAm) triggered by solvent (water and ethanol). Reproduced with permission from John Wiley and Sons [64].

Chemically responsive shape memory polymers undergo shape morphing upon external chemical cues, which mainly comprise solvents, various ions, or pH in a cytocompatible range [24,30]. Similar to thermoresponsive SMHs, several combinations of chemically responsive SMHs are developed by using dual networks. For example, an IPN consisting of a stable network from pluronic F127 diacrylate macromer (F127DA) and a reversible network from alginate sodium alginate was reported to fabricate SMHs for drug delivery using Ca^2+^ as chemical stimuli [56]. Similarly, pH-sensitive IPN-based SMHs are reported from a combination of poly(diacetone acrylamide-co-AAm-co-AA)/alginate (PDAAc-A) by modulation of hydrogen-bonding interactions to exhibit pH sensitivity. These hydrogels were modulated by changing the pH value to 5, as well as adding 1 M of ferric chloride solution [57]. Another interesting approach to developing SM hydrogel actuators was based on P(AAm)-based semi-interpenetrating networks, i.e., P(AAm) and P(AAm-co-MAA) formed in the presence of gelatin. By taking advantage of the coil–triple helix transition of gelatin, the authors programmed the hydrogel actuators at different temperatures (5 °C and 50 °C) that underwent shape-morphing upon actuation at pH 2 (Figure 3A) [58]. In addition to IPNs, the various copolymers exhibit chemoresponsive shape morphing. For example, pH-induced SMHs were reported from a copolymer of AA and AN, where the non-covalent hydrogen bond interactions were modulated by changing the pH. Interestingly, the programming (permanent shape) of these gels was carried out in acidic conditions (1M HCl), and the gel recovery was demonstrated upon exposure to basic conditions (5 wt% sodium bicarbonate solution). The authors further reported that zinc ions can be added to improve the electrical conductivity of these gels [59]. Moreover, the AN-based pH-responsive SMHs were also demonstrated by the copolymerization of AN with N-acryloyl 2-glycine (ACG). In acidic conditions, the rigid copolymeric network consists of protonated carboxyl groups of ACG forming supramolecular hydrogen bonds and cyano groups from AN-forming dipole–dipole interactions. In neutral pH conditions, the strips from P(AN-co-ACG) hydrogel undergo shape morphing, forming micro-coils, and were implicated in the fabrication of microcatheters [60].

In addition to pH modulation, other coordination ionic interactions are also widely employed to control shape morphing of SMHs; for example, a ter-polymer of AAm, AA, and acryl-adamantanamine (Ad-Am) monomers polymerized in the presence of β-cyclodextrin-modified tunicate cellulose nanocrystal (β-CD−TCNCs)-based hydrogels were reported. These gels were programmed through Fe^3+^/−COO^−^ ionic coordination into a variety of shapes (stretching, twisting, and coiling) via host–guest interactions to reinforce the hydrogel and shape recovery in the presence of solvents (ethanol) that cause shrinkage of the network. These hydrogels were later used for the fabrication of tendril-inspired hydrogel artificial muscles (Figure 3B) [61]. In addition to single ions, the polyions can be used as charged polymers; towards this end, the polyion complex (PIC) from pH-responsive hydrophobic 2-(diethylamino)ethyl methacrylate (DEAEMA) in poly(styrene sulfonic acid) was used. This network became hydrophilic in acidic solutions and stabilized in the presence of counter-ion NaCl. This network undergoes pH-induced shape memory behavior where the gels were swollen in HCl while shrinking in NaOH [62]. Similarly, pH-responsive SMHs were fabricated from the polyionic copolymer of 2-(dimethylamino)-ethyl methacrylate (DMAEMA), [3-(methacryloylamino) propyl] trimethylammonium chloride (MPTC), and sodium p-styrenesulfonate hydrate (NaSS). These polyampholyte SMHs undergo shape morphing at a low pH (0.5 M HCl) from a helix form to shape recovery at a high pH (0.5 m NaOH) (Figure 3C) [63].

Recently, hydration has emerged as a widely employed biocompatible cue to tune the shape-memory properties of polymers. In this regard, keratin-based polymer transitions from α-helix to β-sheet as an actuation mechanism. These gels were programmed by shear stress to induce their self-organization into a permanent nematic phase and can transform into anisotropic structuring upon exposure to water [65]. In addition to water, other solvents (e.g., ethanol, DMSO) were also reported for chemoresponsive SMHs. Towards this end, copolymer of P(AAm) and PF127-DA lead to the development of chemoresponsive SMHs. These PF-127-based dynamic micelles containing hydrogels undergo ethanol-induced crystallization while exhibiting cyclical shape recovery in water due to de-crystallization of the copolymerized network (Figure 3D) [64].

### 3.3. Light Responsive SMHs

**Figure 4:**
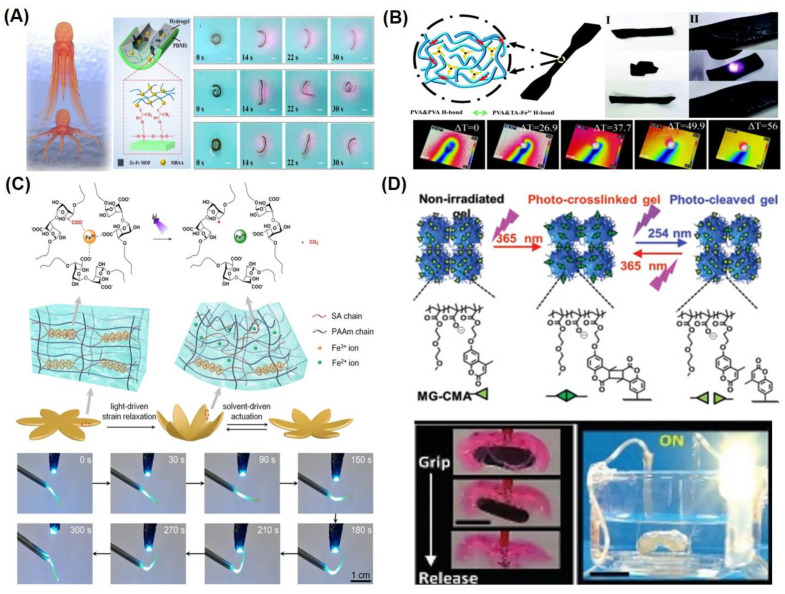
Representative examples of light-responsive SMHs. (**A**) Schematics of the octopus-inspired MOF-loaded P(NIPAM) hydrogel functionalized on 3-(trimethoxysilyl)propyl methacrylate (TMSPMA)-modified PDMS surface (left), with digital camera images of shape-morphing stages of such MOF-containing hydrogel actuators on exposure to 808 nm NIR light (right). Reproduced with permission from the American Chemical Society [70]. (**B**) Schematics of PVA-based hydrogels, crosslinked by Fe^3+^ (top left), with images of the shape recovery process of such PVA/TA-Fe^3+^ hydrogels within 120 s of near-infrared irradiation (top right), and with near-infrared photothermal images of hydrogels under irradiation of 808 nm (bottom). Reproduced with permission from the Royal Society of Chemistry [72]. (**C**) Schematic illustration of the SA/PAAm-based hydrogels. The photo-triggered reductions of Fe^3+^ ions dissociate the SA network (top), with digital camera images of shape-morphing stages of such hydrogels under light irradiation (365 nm). Reproduced with permission from the American Chemical Society [73]. (**D**) Schematic representation of the photo-switching of MG-CMA gel upon UV irradiation, with digital camera images of shape morphing of gripper from such polymer that could release a load underwater on exposure to 254 nm. Reproduced with permission from John Wiley and Sons [74].

Light-responsive shape memory polymers undergo shape morphing upon exposure to UV light; however, in recent years, several studies on near-infrared (NIR)-mediated SMHs have been reported [66,67,68,69]. NIR not only imparts very little phototoxicity but also allows for better tissue penetration, making NIR-based SMHs more attractive for biomedical applications. To this end, an interesting approach is reported for octopus-inspired NIR light-driven SMH actuators using Zr–Fc-based metal–organic frameworks (MOFs). The nanosheets of Zr–Fc MOFs were incorporated as photothermal nanotransducers that, upon exposure to NIR, trigger shape morphing of well-known thermoresponsive poly(N-isopropylacrylamide) P(NIPAM) hydrogels. Upon actuation of Zr–Fc MOF containing P(NIPAM) hydrogels at 808 nm light, the hydrogel actuators undergo bending, while exhibiting fast shape-recovery in the absence of light. Interestingly, upon cyclical NIR illumination of these nanocomposites, the SMHs mimicked the swimming movement of an octopus underwater (Figure 4A) [70]. A similar strategy was adopted for the development of NIR hydrogel actuators using P(NIPAM)-hydrogels pattered with polypyrrole [71]. In addition to photothermal activation, the introduction of Fe^3^ ions is another frequently employed method to synthesize SMHs. Accordingly, NIR hydrogels were fabricated from a PVA and tannic acid (TA)-Fe^3+^ complex. The TA-Fe^3+^ forms an H-bond with PVA, and these complexes allow for photothermal shape memory response upon irradiation with an 800 nm laser for a 120 s duration (Figure 4B) [72].

Apart from NIR-mediated SMHs, traditional UV-actuated SMHs are widely investigated. The double network based on sodium alginate (SA) and P(AAm) hydrogels is formed by Fe^3+^-carboxylate coordination with the alginate network, which undergoes shape morphing due to the light-induced (at 365 nm) reduction of Fe^3+^, causing the softening of the alginate network (Figure 4C) [73]. Another approach for UV-light-driven shape morphing involves the photo-triggered covalent interlinking of coumarin-based microgels (MGs) from a copolymer of 2-(2-methoxyethoxy) ethyl methacrylate, methacrylic acid, and 7-(2-methacryloyloxyethoxy)-4-methylcoumarin [poly(MEO2MA-co-MAA-co-CMA]. These gels were programmed into a temporary shape by photocrosslinking using 365 nm UV light and subsequently photocleaved using 254 nm UV light, and were used for the development of photoswitchable actuators and grippers (Figure 4D) [74]. Stimuli-responsive DNA-based hydrogels, incorporated in the P(AAm) network, are cooperatively crosslinked by glucosamine-boronate esters and duplex nucleic acid bridges stabilizing trans-azobenzene intercalator units bridged by stimuli-responsive nucleic acids. Photoisomerization of trans-azobenzene between UV and visible light to cis-azobenzene states in these stimuli-responsive SMHs leads to switchable stiffness properties of the hydrogel [75]. 

### 3.4. Electrically Responsive SMHs

**Figure 5:**
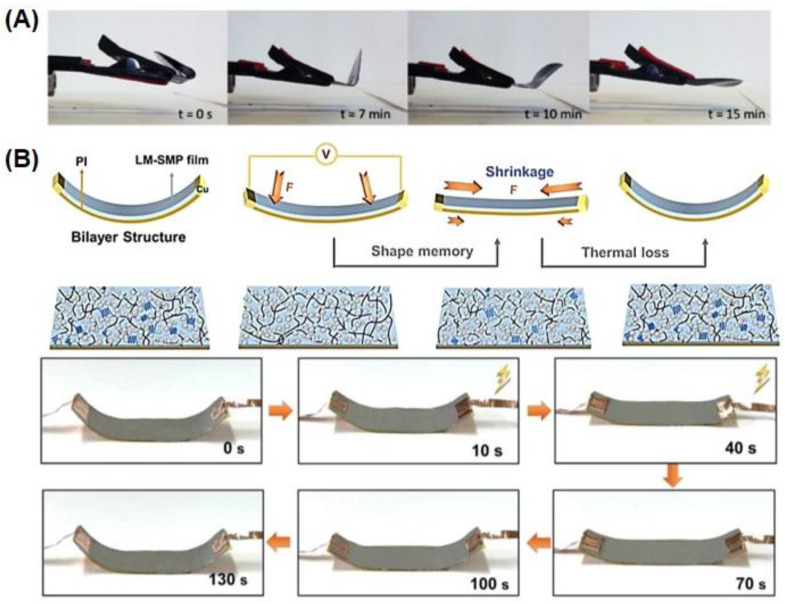
Representative examples of electrically responsive SMHs. (**A**) Digital camera images of shape-morphing stages of polymeric composite of XNBR Krynac containing multiwalled carbon nanotubes (MWCNTs) upon the application of a bias of 50 V. Reproduced with permission from the Multidisciplinary Digital Publishing Institute [80]. (**B**) Schematics showing PCL-based shape memory hydrogels containing liquid metal (LM) (gallium and zinc) to enhance the conductivity (top), with digital camera images of shape-morphing stages of such polymeric films upon the application of a bias of ~2 V. Reproduced with permission from Elsevier [84].

Electrically responsive SMHs are reversibly actuated upon stimulation by an external electric field. The electric field results in a nonuniform distribution of ions that causes osmotic pressure imbalance or Maxwell stress, leading to a bending motion of the hydrogel. Electrically conductive hydrogels are typically prepared by the addition of conductive fillers (e.g., metal oxides, carbon black, and multiwalled carbon nanotube) into the polymeric networks [76,77,78,79]. Towards this end, the elastomer of XNBR Krynac^®^ X 740 (Lanxess Elastomers SAS, Stuttgart, Germany) was incorporated with carbon black and multiwalled carbon nanotubes (MWCNTs) to enhance the conductivity of the hydrogels. These conducting SMHs undergo a shape-memory effect upon the application of bias of 50 V (Figure 5A) [80]. Similarly, poly(ethylene vinyl acetate)/poly(ε-caprolactone) (EVA/PCL) or polyurethane (PU) hydrogel matrices were mixed with CNTS to fabricate conductive SMHs that undergo electrically modulated shape morphing [81]. Moreover, carbon nanotubes (CNT) containing polyvinyl alcohol/chitosan (PVA/Cs) hydrogel networks undergo shape morphing upon electrical actuation at 40 V [36], while CNT containing silk fibroin (SF) also demonstrated electrically regulated shape morphing [82]. In addition to CNTs, graphene oxide (GO) containing IPN of SF with P(AAm) showed high electrical conductivity and shape-morphing properties [83].

The fabrication of electrically responsive biocompatible SMHs from these conductive polymeric nanocomposites is limited by the actuation at high voltages. An interesting approach to fabricating low-voltage electroactive SMHs was found by adding liquid metal (gallium and zinc) into PCL. The resulting LM–SMP film was thermally programmed into a permanent shape and could be electrically actuated at low voltages (~2 V) for electrically driven recovery of LM–SMP (Figure 5B) [84]. Similarly, chitosan-based injectable electroactive SMHs were demonstrated, where pendent groups of chitosan were functionalized with carboxylic Pluronic and aniline pentamers. The self-assembled Pluronic facilitated thermoregulated crosslinking, allowing for the reprogramming of polymers [85]. Upon actuation, such hydrogels allow for the release of the electrically regulated delivery of VEGF. Analogously, conducting PVA-based SMHs were demonstrated by the addition of 5,5,6,6-tetrahydroxy-3,3,3,3-tetramethyl-1,1-spirobisindane (TTSBI)-Fe^3+^ in the PVA network. These SMHs undergo shape morphing in response to multiple stimuli, including temperature, solvent, and Fe^3+^, because of the crystalline domains of the PVA chains, solvent–polymer interactions, and catechol–Fe^3+^ interactions, respectively [86]. 

**Table 1:** Overview of representative state-of-the-art SMHs categorized on the basis of polymeric networks commonly used for forming hydrogels. The fabrication of different types of SMHs developed from such polymers with their underlying shape morphing strategies (i.e., physical crosslinking or modifications) and relevant actuation mechanisms are summarized.

Core Polymeric Hydrogel Network	Shape Memory Hydrogel Matrix	Influence of Additives on Properties or Function of SMHs	Type of Stimuli	Ref.
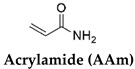	P(AAm-co-PEA)	DMSO/Water (DMSO allowed for hydrophobic interactions for the formation of hard polymer network)	Temperature (37 °C)	[46]
	P(AAm) and ELP	-	Temperature (20 °C)	[47]
	P(AAm-co-MAA)	Gelatin (Thermally induced coil-triple helix transition of gelatin facilitates the welding between copolymers)	Chemical (pH 2)	[58]
	P(AAm-co-AAc-co-Ad-Am)	TCNC nanocrystals and Fe^3+^ ions (TCNC enables the shaping process of hydrogesl while Fe^3+^ ions assists in the fixation of network via Fe^3+^-COO^-^ coordination).	Chemical (EtOH)	[61]
	P(AAm) and SA	Fe^3+^ ions (To help in strain fixation via Fe^3+^-carboxylate coordination)	Light (UV = 365 nm)	[73]
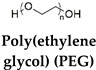	PEG, MDI, and IU	-	Temperature (4°C to 50°C)	[45]
	PEG, MDI, and IU	Tannic acid and Kartogenin (To promote MSC differentiation into chondrocytes)	Temperature (37°C)	[52]
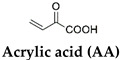	-	-	Temperature (Ambient)	[53]
	P(DA-co-AAm-co-AA) and alginate	-	Chemical (Ferric Chloride)	[57]
	P(AA-co-AN)		Chemical (pH)	[59]
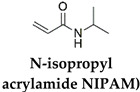	P(NIPAM)	Nanosheets of Zr-Fc MOFs (MOFs function as a photothermal nanotransducer and enhance the actuation performance of the hydrogel actuators)	Light (NIR = 800 nm)	[70]
	P(NIPAM-AA), PPy and alginate	Fe^3+^ ions (To induce the formation of the PPy pattern)	Light (NIR = 800 nm)	[71]
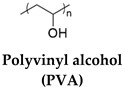	PVA and chitosan	Graphene oxide (It crosslinked with chitosan to form a network of the hydrogel by electrostatic interactions)	Temperature (60°C)	[40]
	PVA and cornstarch	-	Chemical (water)	[48]
	PVA	Tannic acid and Fe^3+^ ions (To endow the hydrogel with a good photothermal effect)	Light (NIR= 808)	[72]
	PVA and chitosan	Carbon Nanotubes (To enhance the mechanical and electrical properties of the hydrogel system)	Electricity (40 V)	[36]
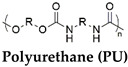	-	-	Temperature (10 °C)	[43]
	LBL assembly of PU with CHTMA, LAMMA, or HAMA, GELMA.	-	Temperature (37 °C)	[44]
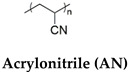	P(AN-co-AAm)	BaSO_4_ (To equip the hydrogel coils with radiopacity)	Temperature (20 to 40 °C)	[51]
	P(AN-co-ACG)		Temperature/Chemical (37 °C and pH 6)	[60]
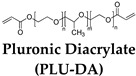	PLU-DA and SA	-	Chemical (Ca^2+^)	[56]
	(PLU-DA-co-AAm)	-	Chemical (Ethanol)	[64]
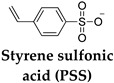	P(SS-DMAEMA-co-MPTC)	-	Chemical (pH)	[63]
	P(SS-co-DEAEMA)	-	Chemical (pH)	[62]
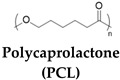	PCL and PEG	DA-Diols (Act as a chain extender and initiate thermally driven retro Diels–Alder reaction)	Temperature (37 °C)	[50]
	-	Liquid Ga and Zn metal (To enhance electrical conductivity and facilitate electricity induce shape deformability)	Electrical (2 V)	[84]

AAm = acrylamide, PEA = 2-phenoxyethyl acrylate, ELP = elastin-like polypeptides, Ad-Am = Acryl-adamantanamine, MAA = methacrylic acid, NIPAM = N-isopropylacrylamide, PVA = Polyvinyl alcohol, SA, ACG = N-acryloyl 2-glycine, CHTMA = methacrylated chitosan, LAMMA = methacrylated laminarin, HAMA = methacrylated hyaluronic acid, GELMA = methacrylated gelatin, MDI = methylenediphenyl 4, 4- diisocyanate, IU = imidazolidinyl urea, DA = diacetone acrylamide, DEAEMA = 2-(diethylamino)ethyl methacrylate, DMAEMA = P(SS-2-(dimethylamino)-ethyl methacrylate, MPTC = [3-(methacryloylamino)propyl]trimethylammonium chloride.

**Table 2:** An overview of the processing of different representative SMHs into functional responsive interfaces.

Type of SMHs	Fabrication Strategy for Development of SMHs	Applied Stimuli	Application of SMHs	Ref.
Thermally Responsive	Copolymer of P(AAm-co-PEA) physically stabilized by DMSO-mediated hydrophobic interactions	37 °C	Reconfigurable Surgical Scaffold	[46]
	Physically crosslinked dual networks of P(AAm) and ELP	20 °C	Hydrogel Actuators	[47]
	Dual-network of PCL and PEG crosslinked by retro Diels–Alder reactions	37 °C	Implantable Scaffold	[50]
	Copolymer of P(AN-co-AAm) physically crosslinked with BaSO_4_	40 °C	Reconfigurable Surgical scaffold	[51]
	Copolymer of P(AN-co-ACG) physically crosslinked by supramolecular hydrogen bonds	37 °C	Implantable Scaffold	[60]
Chemically Responsive	IPNs of PLU-DA and SA physically crosslinked by Ca^+2^ ions	Calcium Ions	Drug Delivery	[56]
	IPNs of P(DA-co-AAm-co-AA) and alginate, physically crosslinked by Fe^+3^/COO^-^ interactions	Ferric Ions	Hydrogel Actuators	[57]
	Copolymer of P(SS-co-DMAEMA-co-MPTC), physically crosslinked by polyionic interactions	pH	Soft Robotics	[63]
Photo-responsive	Nanocomposite of P(NIPAM) containing Zr-Fc MOFs, which act as photothermal nanotransducers	800 nm	Hydrogel Actuators	[70]
	IPNs of P(NIPAM-AA), PPy and alginate, physically crosslinked by Fe^+3^/COO^-^ interactions	800 nm	Hydrogel Actuator	[71]
Electrically Responsive	Dual networks of PVA and chitosan, containing CNTs to enhance electrical conductivity	40 V	Hydrogel Actuator	[36]
	PCL containing gallium and zinc metal to enhance electrical conductivity and responsivity	Electrical (2 V)	Hydrogel Actuator	[84]

## 4. Biomedical Applications of Shape Memory Hydrogels

The ability of SMHs to change their shape, size, structure, and physicochemical properties (wettability, mechanical and optical properties) makes them exceedingly useful for various biomedical applications, such as surgical implants, modulated drug delivery, artificial muscles, tactile interfaces, and biosensing. The reversible shape morphing of SMHs not only allows for mimicking the dynamics of biological tissues but also offers adaptability under changing external conditions [87,88,89,90]. 

### 4.1. SMHs for Tissue Regeneration Therapies

**Figure 6:**
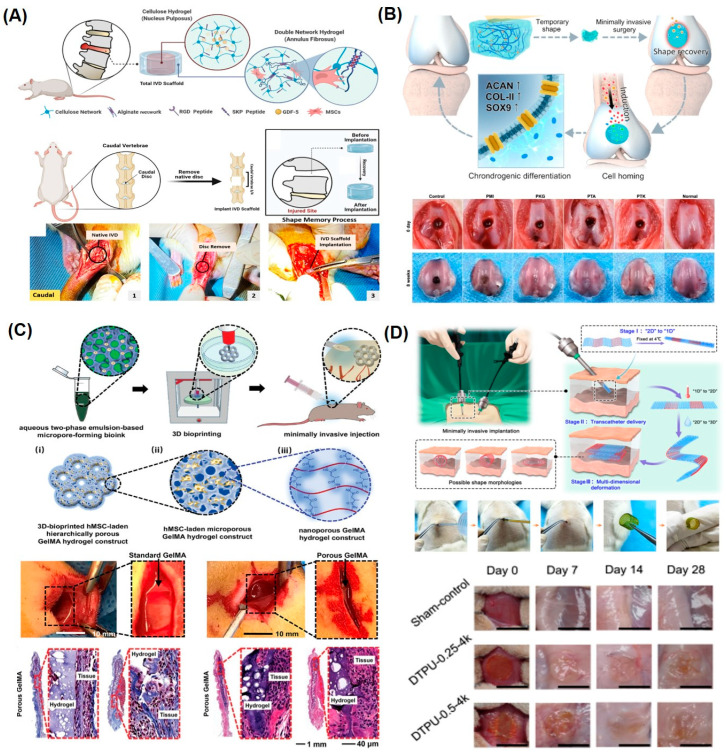
Representative examples of SMHs for tissue regeneration therapies. (**A**) Schematics of implantation of IVD scaffolds into the rat caudal spine to replace the native caudal disc, fabricated from double network hydrogels via physically crosslinking alginate network and chemically crosslinking cellulose network (top), with digital camera images of surgical implantation of IVD scaffolds into the rat caudal spine. EDTA triggered the shape recovery process of double network hydrogel in the caudal disc of the rat (bottom). Reproduced with permission from John Wiley and Sons [93]. (**B**) Schematic demonstration of dynamic hydrogen bond containing a crosslinked polymeric network based on PU, PEG, IU, and MDI. These SMHs were further loaded with TA and KGN to promote the differentiation of BMSCs into chondrocytes for cartilage regeneration (top), with digital camera images of femur condyles of SD rats after 8 weeks of surgical implantation of such hydrogels. In the SMH-treated group, the regenerated hyaline-like cartilage tissue with smooth surfaces exhibited intact connection with adjacent normal cartilage without obvious boundaries, which was similar to the normal cartilage tissue. Reproduced with permission from Springer Nature Publishing Group [52]. (**C**) Schematic overview showing the fabrication process of the injectable porous gelatin methacryloyl (GelMA) hydrogel constructs and minimally invasive injection using a commercial percutaneous needle (top), digital camera images show the integration of micro–nanoporous hydrogel constructs into the defect areas of SD rats after 2 weeks, and Masson’s trichrome staining and H&E staining of the porous GelMA hydrogel at 1 week and 2 weeks post-implantation (bottom). Reproduced with permission from John Wiley and Sons [14]. (**D**) Schematics of the delivery of the dynamic thermoset polyurethanes (DTPU) scaffolds in a minimally invasive manner through multidimensional morphing (1D to 3D) triggered by body temperature (top), with digital camera images of implanted 4D printed scaffolds to complete 1D to 3D deformation subcutaneously in SD rats, with images of implanted DTPU scaffolds in rats at different time intervals (Bottom). Reproduced with permission from Springer Nature Publishing Group [50].

The biocompatibility, tunable mechanical properties, inhibition of non-specific cell attachment, and selective biofunctionalization of SMHs are key features to employ for tissue regeneration therapies [20,91,92]. In addition to shape morphing, the SMH-based tissue engineering scaffold can facilitate the release of specific bioactive biomolecules. Towards this end, an SMH-based intervertebral disc (IVD) scaffold was reported from a physically crosslinked highly compressible double network of alginate and cellulose network to treat IVD degeneration. The alginate network was molded into a cylindrical shape, forming the annulus fibrosus (AF), and a cellulose solution was employed to fabricate the nucleus pulposus (NP). The alginate network was functionalized with cell-adhesive peptide (GRGDSP), while the cellulose network was functionalized with homing peptide (SKPPGTSS) for MSCs; Ca^2+^ ions were utilized to fix the temporary shape. The constructed IVD scaffolds were implanted into the rat caudal spine to replace the native caudal disc by taking advantage of the EDTA-based shape morphing of these hydrogels (Figure 6A). Moreover, this scaffold mimics the native IVD structure and controls the delivery of Growth Differentiation Factor-5 (GDF-5), which induces the differentiation of endogenous mesenchymal stem cells (MSCs) [93]. Similarly, injectable SMHs based on Pluronic–chitosan and aniline pentamer were injected in rat models to repair the hippocampus defect due to ischemia. These VEGF-loaded electroactive hydrogels demonstrate reduced infarction volume and improved hippocampal-dependent learning and memory performance in the hippocampus ischemia rat model [85]. Analogously, a scaffold for cartilage regeneration was fabricated from a dynamic hydrogen bond containing a crosslinked polymeric network based on PU, PEG, IU, and MDI. The SMHs were further loaded with tannic acid (TA) and kartogenin (KGN) to promote the differentiation of BMSCs into chondrocytes for cartilage regeneration. The implanted hydrogel scaffold was developed by fixing the temporary shape of the hydrogels by passing through a syringe at 4 °C to facilitate implantation, and these SMHs underwent shape recovery under physiological conditions (37 °C, 95% humidity) in vivo to fill the defect and promote bone regeneration (Figure 6B) [52].

By taking advantage of shape-morphing memory, SMHs are widely explored for the development of reconfigurable scaffolds. For example, gelatin-based SMHs were prepared from an aqueous two-phase emulsion bioink containing the pre-gel gelatin methacryloyl (GelMA)/cell and poly(ethylene oxide) (PEO) blend. The gelatin methacryloyl (GelMA)-based injectable macro–micro–nanoporous cell-laden GelMA hydrogel was constructed through 3D extrusion bioprinting. In contrast to standard GelMA, the microporous hydrogels show better mechanical properties and recovery. The micro–nanoporous hydrogel constructs were subcutaneously injected into Sprague Dawley (SD) rats and evaluated for tissue ingrowth and degradation for up to 2 weeks (Figure 6C). The implanted hydrogel constructs underwent a reduction in the bulk sizes, which, upon the removal of stress, quickly recovered to their original shape at a relaxed state without structure damage and support regeneration [14]. Similarly, the thermally responsive implantable scaffold was developed from PEG and PCL to form a highly swellable 2D printed polymeric mesh, while a low swellable polymeric mesh was fabricated by incorporation of Diels–Alder (DA) diols. These networks were programmed to deform in water forming a 1D mesh while undergoing shape recovery at body temperature (37 °C). The 1D rod was subcutaneously implanted in Sprague Dawley rats, which, within 3 min at body temperature, underwent shape morphing allowing for extension into a 4D scaffold supporting the growth and attachment of cells (Figure 6D) [50].

Due to tunable mechanical properties, the personalized implantable SMH-based scaffolds hold great significance for bone regeneration. In turn, the collagen-based SMHs exhibited outstanding chondrogenic activities and physical properties similar to a typical alginate construct in terms of water absorption, compressive properties, and shape-memorability behavior. The treatment of the rat tail nucleotomy model with shape memory collagen-cryogel/HA alleviated mechanical allodynia, maintained a higher concentration of water content, and preserved the disc structure by restoring the matrix proteins [94]. Similarly, the silk fibroin-based cryogels were employed as implantable scaffolds for osteogenic differentiation and bone marrow recovery. These SF hydrogels were combined with laponite (LAP) nanoparticles and programmed into a temporary shape by freezing. Upon subcutaneous injection of the SF-MA/LAP scaffold in Balb/c mice, the cryogels underwent rapid volumetric recovery after injection through a needle at body temperature, with enhanced proliferation, spreading, and osteogenic differentiation of the seeded bone marrow-derived mesenchymal stem cells (BMSCs) [95].

Interestingly, a polyurethane-based implantable multisite optogenetic stimulation device (MOSD) was fabricated for neural stimulation. For site-selective stimulation of sciatic nerve bundles, the PU-based thermoresponsive MOSD was implanted in mice with severed and anastomosed nerves to restore selective limb movement [43]. Similarly, thiolene/acrylate-based thermoresponsive SMHs were employed to fabricate a double-clip neural interface (DCNI). The authors implanted fabricated DCNI on a small branch of the sciatic nerve in a rat model for electrical stimulation and electroneurography (ENG) recording. The results demonstrate that the proposed neural interface can be used for the modulation of the peripheral nerve, including the autonomic nerve, towards bioelectronic medicine [49].

### 4.2. SMHs for Surgical Applications

**Figure 7:**
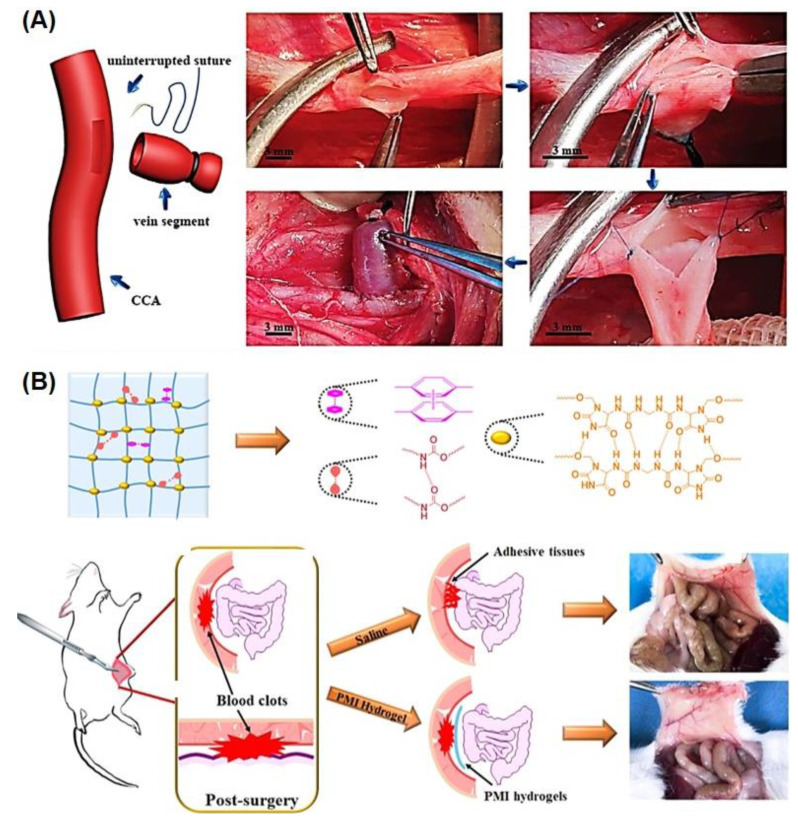
Representative examples of SMHs for surgical applications. (**A**) Schematic illustration of insertion of dual temperature/pH responsive micro-coil fabricated from P(AN-ACG) SMHs in a dog’s carotid artery (CCA) side-wall aneurysm model (top), with digital camera images of the process, where such a microcoil underwent shape morphing in the neural pH of the blood microenvironment to enhance the packing density and reduce recanalization for the treatment of intractable aneurysms (bottom). Reproduced with permission from John Wiley and Sons [60]. (**B**) Schematics for the preparation of the PMI hydrogels containing hydrogen bonds among imidazolidinyl urea (IU), urethane groups, and hydrophobic interaction of the benzyl ring of MDI (top), with digital camera images of the antiadhesion effect of such hydrogels in a mouse’s abdominal wall defect (bottom). Reproduced with permission from the American Chemical Society [45].

The SMHs are finding great traction for surgical procedures, especially as stents and catheters [92,96]. An interesting approach was to develop soft catheters, e.g., to treat vascular disease. Towards this end, radiopaque SMHs were fabricated by copolymerizing acrylonitrile (AN), and a pH-responsive N-acryloyl 2-glycine (ACG) exhibited a body temperature-triggered SM effect. These stiff SMHs were transported into a dog’s carotid aneurysm sac, where the hydrogels underwent shape morphing in the neural pH of the blood microenvironment to enhance the packing density and reduce recanalization for treatment of intractable aneurysms (Figure 7A) [60]. Similarly, body-temperature-triggered SMHs were fabricated using reversible hydrophobic dipole-pairing microdomains in the flexibly crosslinked network of P(AAm) (acting as a hydrogen-bonding monomer), acrylonitrile (AN) (acting as a dipole monomer), and a long flexible PEG-MA crosslinker, followed by solidification by using BaSO_4_. These hydrogels demonstrated interesting mechanical properties comparable to those of rubber and underwent reversible shape morphing by adjusting temperatures ranging from 20 °C to 40 °C, which allows for their use in transcatheter arterial embolization [51]. Moreover, postoperative antiadhesion physical barriers were developed from PEG-based SMHs by polyaddition polymerization with methylenediphenyl 4, 4-diisocyanate (MDI), and imidazolidinyl urea (IU). These supramolecular hydrogels match the mechanical properties of human soft tissues (smooth muscle) and were programmed into a temporary shape at 4 °C. Upon implantation of these antiadhesion thermoresponsive SMHs into the abdominal cavities of mice, these hydrogels provided postoperative anti-adhesion physical barriers and prevented peritoneal adhesion after abdominal or pelvic procedure (Figure 7B) [45].

## 5. Conclusions and Future Directions

SMHs are promising materials for biomedical applications because of their biocompatibility, wettability, high stretchability, antifouling properties, and selective biofunctionalization. In contrast to traditional stimuli-responsive polymers, shape-memory materials not only recover to designed dimensions but also exhibit shape morphing at a relatively faster rate. Despite the excellent potential of stimuli-responsive wearables and implantables, access to external stimuli (light, heat, electricity) in complex biological systems is challenging. With recent advancements in the design and engineering of SMHs, the paradigm is shifting towards the development of SMHs with easily accessible stimuli (e.g., body temperature, natural enzymes, body pH, or electrolytes). These naturally triggered SMHs with a tunable recovery as well as potential biodegradability can solve the bottlenecks in the development of dynamic biomedical devices.

Furthermore, SMHs exhibit shape-morphing behavior due to the dynamic properties of a bulk polymeric matrix comprising multiple copolymers or IPNs. However, current shape-memory polymeric materials lack long-range molecular order that enables more controlled and efficient actuation mechanisms. This is especially relevant for the development of electrically responsive SMHs, where existing electrically responsive SMHs require a significantly high power consumption, low flexibility, poor deformation ability, and high actuation voltages. Consequently, the fundamental understanding of the chemistry of SMHs will allow for the engineering of shape-morphing behavior at the molecular level, such as with the incorporation of dynamic covalent intramolecular interactions. The exploration of polymers with reversible reconfiguration in combination with precise control over their chemical composition and their tunable physicochemical properties are some of the key features that are associated with the development of intrinsically responsive SMHs. 

In recent years, although the area of SMHs has been strenuously explored and flourished for biomedical applications, there is plenty of space available for new ideas and interdisciplinary research—in particular, to develop SMHs for controlled drug delivery and biosensing. Moreover, the incorporation of multi-stimuli-responsive shape morphing and stepwise reconfigurable SMHs are primarily undiscovered. A shift from the mere proof-of-concept stage to real biomedical applications is expected in the near future.
